# Diagnostic value of cervical vertebral maturation stages for midpalatal suture maturation assessment: a study in the Chinese population

**DOI:** 10.1186/s12903-023-03220-7

**Published:** 2023-07-20

**Authors:** Henglang Liu, Linjing Feng, Lili Wang

**Affiliations:** 1 Department of Stomatology, Sichuan Academy of Medical Sciences & Sichuan Provincial People’s Hospital, 32# W. Sec 2, 1st Ring Rd., Chengdu, Sichuan Province 610072 China; 2 Chengdu Stomatological Hospital, No.17 Chunxi Road South, Chengdu, Sichuan Province 610000 China; 3 School of Medicine, University of electronic science and technology of china, No.4, Section 2, North Jianshe Road, Chengdu, 610054 China

**Keywords:** Midpalatal suture, Cervical vertebrae, Maturation stage, Diagnostic test

## Abstract

**Purpose:**

To evaluate the correlation between cervical vertebral maturation stages (CVMS) and midpalatal suture maturation stages (MPSMS), and to analyze the diagnostic value of CVMS for the assessment of MPSMS.

**Methods:**

Cone beam computed tomography (CBCT) images of 233 subjects (8–20 years) were selected. The CVMS was determined using the McNamara and Franchi method, while the MPSMS was evaluated using the Angelieri method. Spearman rank correlation was used to analyze the results, and positive likelihood ratios were calculated to evaluate the diagnostic value of CVMS in identifying MPSMS.

**Results:**

Spearman rank correlation results showed a strong positive correlation (*r* = 0.867, *P* < 0.001) between CVMS and MPSMS. The positive likelihood ratios of CS12, CS4, and CS56 for the identification of stages AB, C, and DE were 12.17, 7.64, and 7.79, respectively. The values of the positive likelihood ratios of the other groups were less than five.

**Conclusion:**

CS12 of the CVMS can be used as a reliable indicator for the assessment of MPSMS stage AB. From CVMS stage 4 forward, midpalatal suture maturation should be evaluated using CBCT.

## Introduction

Rapid Maxillary Expansion (RME) is a primary treatment for transverse maxillary deficiency. Prior to RME treatment, evaluating midpalatal suture maturation may provide valuable guidance for clinical treatment planning [1]. If the midpalatal suture is fully fused, expanding the arch blindly may cause dental expansion to exceed skeletal expansion, which can result in unintended side effects [2], such as iatrogenic inclination of the supporting teeth, gingival recession, and damage to the periodontal tissues. Midpalatal Suture Maturation Stages (MPSMS) is a classification method that defines the level of maturation of the midpalatal suture and requires high-quality cone-beam computed tomography (CBCT) images for identification [3]. As a component of the maxilla, the maturational stages of the midpalatal suture's development are naturally linked to the individual's growth and development. Meanwhile, the Cervical Vertebral Maturation Stages (CVMS) is a useful biological indicator for studying the pubertal peak and further development of skeletal growth [4, 5]. Unlike MPSMS, the CVMS can be assessed using lateral cephalograms, which are commonly used for orthodontic diagnosis and treatment planning [6]. If clinicians can indirectly predict MPSMS based on a patient's CVMS with reasonable accuracy, this will benefit clinical applications. To date, there have been a limited number of studies investigating the correlation between CVMS and MPSMS, such as Angelieri [7] et al. Therefore, we aim to investigate the diagnostic value of CVMS in predicting MPSMS in the Chinese population.

## Method

### Subjects

We enrolled patients who were evaluated at the Department of Stomatology, Sichuan Academy of Medical Sciences & Sichuan Provincial People's Hospital between 2019 to 2022. The radiographic images were obtained for treatment purposes. Inclusion criteria and exclusion criteria were as follows:

### Inclusion criteria

I) age between 8–20 years; II) CBCT image field of view that covered at least the intact hard palate to the inferior borders of the fourth cervical vertebrae; III) no systemic diseases affecting bone metabolism; IV) no history of cleft lip or palate treatment; V) no history of orthodontic or orthognathic surgery.

### Exclusion criteria

I) presence of supernumerary or impacted teeth in the midpalatal suture area; II) substandard resolution or quality of CBCT imaging, which interfered with the identification of anatomical structures.

### Instruments and software

This study utilized a CBCT machine (Vatech, South Korea) for image acquisition. During the scanning process, patients were required to maintain the Frankfort plane parallel to the ground, while their chin was placed on the chin rest. The following scanning parameters were used: 85 kVp, 10 mAs, and 12-s scan time. The acquired DICOM files were reconstructed using Ez3D Plus imaging software, which automatically derived lateral cephalograms from the same CBCT images.

### Evaluation method of CVMS and MPSMS

For the analysis of CVMS, the second, third, and fourth cervical vertebrae images were obtained from the lateral cephalograms. The morphology of the cervical vertebrae was determined using the McNamara and Franchi method [8]. CVMS was categorized into six different stages, as shown in Table 1.

**Table 1:**
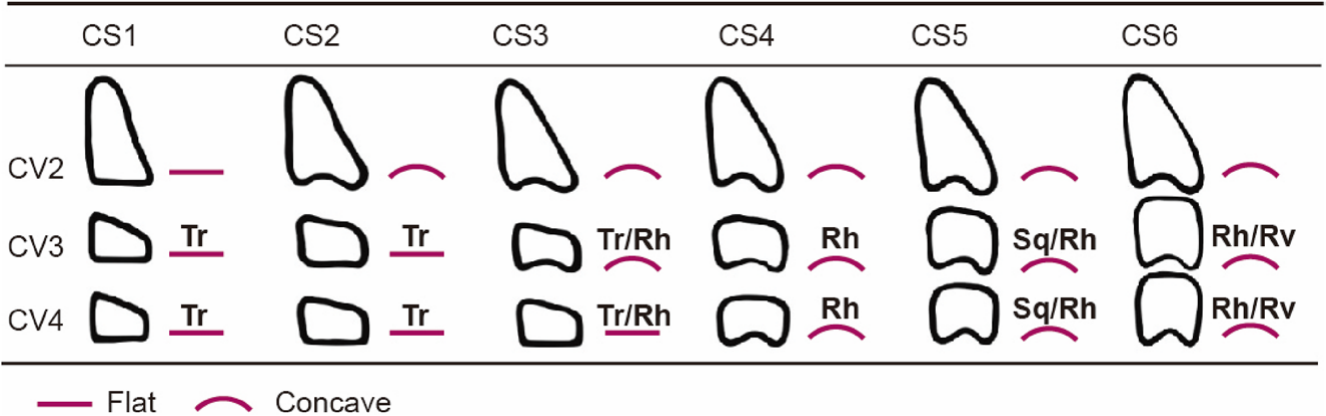
Cervical vertebral maturation stages according to the method of McNamara and Franchi

*Abbreviations*: *Tr* trapezoidal, *Rh* rectangular horizontal, *Sq* square, *Rv* rectangular vertical

**Fig. 1:**
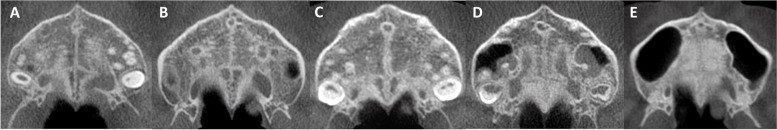
Maturation stages **A**, **B**, **C**, **D** and **E** of the midpalatal suture. Stage **A** is characterized by a approximately straight high-density line (unfused suture). Stage **B** is characterized by a curved high-density line (unfused suture). Stage **C** is characterized by two curved line parallel to each other (the beginning of suture fusion). Stage **D** is characterized by two curved lines in the front of the palate that disappeared towards the back (partial suture fusion). Stage **E** is characterized by the completely disappear of the suture line (complete suture closure)

To evaluate MPSMS, high-quality CBCT images were used to identify the level of midpalatal suture maturation. The Angelieri [3] method was used, which involved overlapping the midpalatal suture simultaneously with the vertical reference lines in the axial and coronal view. Next, a horizontal reference line was passed through the center of the cancellous bone of the hard palate in the sagittal view. An axial view image of the palatal suture was then obtained for analysis. The MPSMS classification system was used to categorize midpalatal suture maturation into five stages, as in Fig. 1.

### Method for image staging assessment

Each image in the study sample is assigned a unique identification number. A researcher who is proficient in both evaluation methods sequentially interprets the CVMS and MPSMS of each sample.

After one month interval, 30 images were randomly selected from the entire sample and re-evaluated by the same evaluator. The degree of intra-examiner agreement was determined using a weighted kappa coefficient.

### Method of evaluation

The cervical stages CS1 and CS2 were combined into CS12 (prepubertal stage), and CS5 and CS6 were combined into CS56 (postpubertal stage) for practical clinically purposes. CS3 and CS4 were analyzed separately. To further align with clinical practice, we reconsolidated the midpalatal suture maturation stages based on the fusion of the midpalatal suture. We combined stage A and stage B into stage AB (no fusion of the midpalatal suture), and stage D and stage E into the stage DE (partial or complete fusion of the midpalatal suture). We analyzed stage C separately as a transitional stage, as it implies the begining of fusion of the midpalatal suture.

The positive likelihood ratio (LHR) was used as a measure of diagnostic performance to assess the relationship between CVMS and MPSMS. For each CVMS stage (CS12, CS3, CS4, and CS56), we created a four-grid table that combined the different MPSMS stages (AB, C, and DE). The positive likelihood ratio reflected the accuracy of the CVMS in predicting the MPSMS. A higher ratio indicated a higher diagnostic value, indicating that the predicted MPSMS through CVMS was more accurate. A positive LHR ≥ 10 indicated a high diagnostic value, a ratio between five to ten indicated a medium diagnostic value, and a ratio less than two indicated no diagnostic value [9]. Combinations with a positive likelihood ratio greater than five were analyzed in detail to determine their diagnostic value. Specifically, the sensitivity, specificity, negative likelihood ratio, positive predictive value, negative predictive value, and area under the ROC curve (AUC) were calculated for further analysis.

### Statistical analysis

We used SPSS 22.0 for statistical analysis. We used the consistency (Kappa) test to assess the reliability of the repeated interpretation results of CVMS and MPSMS, and the Spearman rank correlation test to measure the correlation between the CVMS and MPSMS. We used the MedCalc 19.2 software to compute various diagnostic efficacy evaluation indices for the CVMS in MPSMS diagnostic tests. The significance level was set at ɑ = 0.05 (two-tailed).

## Results

### Sample characteristics

The study enrolled 223 individuals aged 8 to 20 years, consisting of 92 males (mean age 13.79 ± 3.63 years) and 131 females (mean age 13.51 ± 3.18 years). The CVMS and MPSMS observed in the sample are shown in Table 2.

**Table 2:** Distribution of samples based on CVMS and MPSMS

Age	n	CVMS (n)						MPSMS (n)				
		CS1	CS2	CS3	CS4	CS5	CS6	A	B	C	D	E
8–10	45	26	14	3	1	1		15	20	8	2	-
11–13	70	-	17	17	10	21	5	2	18	23	19	8
14–16	56	-	2	4	12	22	16	-	4	21	11	20
17–20	52	-	-	-	7	26	19	-	5	7	20	20
Total	223	26	33	24	30	70	40	17	47	59	52	48

-: N/A

### Intra-examiner consistency

The twice-evaluated results of CVMS and MPSMS had weighted kappa coefficients of 0.952 and 0.933, respectively. Both coefficients were greater than 0.9, which indicates high intra-examiner reproducibility.

### Correlation analysis between CVMS and MPSMS

The Spearman rank correlation test revealed a statistically significant correlation between CVMS and MPSMS (*r* = 0.867, *P* < 0.001). The correlation remained significant when stratifying the results by gender, with r-value of 0.887 for males and 0.831 for females. There was no significant difference in the correlation coefficient between males and females (Z = 1.5674, *P* > 0.05), indicating no need for gender subgroup analysis.

Table 3 presents the distribution of MPSMS across the different stages of CVMS. The results indicate a gradual maturation and fusion of the midpalatal suture (stages A to E) as the stage of the cervical vertebrae increases (CS1 to CS6).

**Table 3:** Distribution of the MPSMS according to CVMS (n/%)

CVMS	MPSMS					
	A	B	C	D	E	Total
CS1	14/53.8	10/38.5	2/7.7	-	-	26
CS2	3/9.1	22/66.7	8/24.2	-	-	33
CS3	-	10/41.7	14/58.3	-	-	24
CS4	-	3/10.0	22/73.3	4/13.3	1/3.3	30
CS5	-	2/2.9	12/17.1	39/55.7	17/24.3	70
CS6	-	-	1/2.5	9/22.5	30/75.0	40
Total	17	47	59	52	48	223

-: N/A

### Analysis of the diagnostic efficiency of CVMS for MPSMS

Table 4 presents the positive LHR for each CVMS stage (CS12, CS3, CS4, and CS56) corresponding to different MPSMS stages (AB, C, and DE).

**Table 4:** Positive likelihood ratios of CVMS for the diagnosis of MPSMS

CVMS	MPSMS		
	AB	C	DE
CS12	12.17^a^	0.57	-
CS3	1.78	3.89	-
CS4	0.34	7.64^b^	0.25
CS56	0.05	0.37	7.79^b^

^a^positive LHR ≥ 10, ^b^positive LHR ≥ 5, - N/A

Three combinations showed a statistical significant correlation between CVMS and MPSMS: CS12 and stage AB (LHR = 12.17, ≥ 10); CS4 and stage C (LHR = 7.64, ≥ 5), and CS56 and stage DE (LHR = 7.79, ≥ 5). Further analysis details for the performance evaluation indicators of these three groups are presented in Table 5. The positive LHR of the other combinations was less than five.

**Table 5:** Diagnostic performance parameters of CVMS for the diagnosis of MPSMS

Parameters	Groups		
	CS12 & AB	CS4 & C	CS56 & DE
Sensitivity (95%CI)	0.77 (0.64–0.86)	0.37 (0.25–0.51)	0.95 (0.89–0.98)
Specificity (95%CI)	0.94 (0.89–0.97)	0.95 (0.91–0.98)	0.88 (0.81–0.93)
AUC (95%CI)	0.85 (0.80–0.90)	0.66 (0.60–0.72)	0.91 (0.87–0.95)
Positive likelihood ratio (95%CI)	12.17 (6.58–22.52)	7.64 (3.60–16.22)	7.79 (4.84–12.54)
Negative likelihood ratio (95%CI)	0.25 (0.16–0.39)	0.66 (0.54–0.81)	0.06 (0.02–0.13)
Positive predictive value (95%CI)	0.83 (0.73–0.90)	0.73 (0.56–0.85)	0.86 (0.80–0.91)
Negative predictive value (95%CI)	0.91 (0.86–0.94)	0.81 (0.78–0.84)	0.96 (0.90–0.98)

*AUC* area under the ROC curve, *CI* confidence interval

## Discussion

The maturation stages of the midpalatal suture are crucial in achieving desirable RME treatment outcomes, which require adequate bony expansion while minimizing dental arch expansion to avoid excessive buccal inclination of anchored teeth. The efficacy of RME is widely believed to be closely associated with the patient's growth and development stage [10]. Baccetti et al. [11] found significant differences in the prognosis of RME treatment among patients at different CVMS stages. However, skeletal age is not a direct reflection of the midpalatal suture development but serves as an indirect prediction of RME prognosis.

CBCT is an effective way to directly assess the maturation stages of the midpalatal suture. Angelieri et al. [3] analyzed CBCT images of the midpalatal suture based on histological research and proposed the staging method of MPSMS. This method is intuitive, effective, and reliable [12] and has been widely used in related studies [13–16], confirming the clinical application value of CBCT in assessing midpalatal suture development. However, considering the radiation exposure of CBCT, its indications should be strictly controlled in clinical practice. Therefore, understanding the correlation between CVMS and MPSMS may help physicians use CBCT more specifically to assess midpalatal suture maturation stages.

The analysis of the subjects revealed that CVMS and MPSMS were highly positively correlated (*r* = 0.867, *P* < 0.01), indicating a close relationship between midpalatal suture maturation stages and skeletal age. This finding is consistent with those of Angelieri et al. [7] and Jang et al. [17]. However, Mahdian et al. [18] reported only a moderately positive correlation between CVMS and MPSMS, which may be due to differences in inclusion criteria. They did not include samples from CS1 and CS2 stages, whereas we included all CVMS stages (CS1 to CS6).

The distribution of MPSMS in each stage of CVMS (Table 3) shows that as the midpalatal suture develops from stage A to E, the maturation of cervical vertebrae increases from CS1 to CS6, consistent with the law of growth and development. Stage D (partial suture fusion) and stage E (complete suture closure) first appeared in CS4 of CVMS in this study. Similar findings were reported in other studies [7, 17, 18]. To further understand the diagnostic value of CVMS staging in inferring specific MPSMS staging, diagnostic performance measures are needed.

To explore whether CVMS and MPSMS have an one-to-one corresponding diagnostic value, we used the positive likelihood ratio as a measurement index. The positive LHR is stable and simultaneously reflects the characteristics of sensitivity and specificity without being affected by prevalence. It is a relatively independent diagnostic test evaluation index. For convenient clinical application, the cervical stages CS1 and CS2 were combined into CS12 (prepubertal stage), and CS5 and CS6 were combined into CS56 (postpubertal stage). CS3 and CS4 were analyzed separately. Similarly MPSMS was consolidated into stages AB, C, and DE. According to Table 4, the meaningful diagnostic positive LHR was more than five in three combinations, CS12 vs. AB, CS4 vs. C, and CS56 vs. DE. The positive LHR of other groups was less than five, indicating little to no diagnostic value [9].

In Table 5, among those groups where the positive LHR was greater than five, we observed the following: Firstly, the positive LHR of CS12 to stage AB was the highest at 12.17 (≥ 10)). This finding suggests that if the CVMS stage is CS12, the probability that the MPSMS stage is AB (unfused suture) is higher. Therefore, CS12 can serve as a reliable indicator to predict stage AB, which supports the conclusion of Baccetti et al. [11] that RME before the growth peak can achieve greater skeletal expansion effects. As a result, it is unnecessary to perform a CBCT examination to determine the midpalatal suture maturation stage for patients with CS1 and CS2.

Secondly, CS4 was not an outstanding diagnostic evaluation indicator for stage C, which could be attributed to the dispersed distribution of MPSMS in CS4, as shown in Table 3. In stage CS4, although stage C accounted for the largest proportion (73.3%), stage D and stage E also appeared in the CS4, accounting for 13.3% and 3.3%, respectively. Therefore, when planning treatment for patients in CS4, we recommended using CBCT to further clarify the midpalatal suture maturation stages.

Thirdly, the negative LHR of CS56 to the DE stage was the lowest at 0.06 (< 0.1),with a negative predictive value of 0.96 and an AUC value of 0.91. This finding indicates that if a patient is not in CS56, the probability of MPSMS being in the stage DE (partial or complete fusion of the midpalatal suture) is low. Therefore, this combination presents a diagnostic value of exclusion [9]. The positive LHR of this combination was less than ten (7.79), indicating moderate diagnostic value. This result may also be related to the dispersed distribution of MPSMS (Table 3). At stage CS5, 2.9% of patients were remained classified as stage B, while 17.1% were classified as stage C. Notably, even one patient at stage CS6 was classified as stage C of MPSMS. These findings could potentially explain the reported successful cases of non-surgical RME treatment in young adults [19]. Moreover, this result suggests that the midpalatal suture may not completely have fused in a small number of patients in CS5 and CS6. Therefore, doctors are recommended to use CBCT examination to avoid over-treatment of patients due to premature use of surgically assisted arch expansion. CBCT examination can also more accurately distinguish whether a patient is in stage D (partial suture fusion) or E (complete suture closure), enabling doctors to create more targeted treatment plans.

In the study by Angelieri [7] et al., the positive LHR of CS3 to stage C indicated high diagnostic value. In contrast, our study found a result of 3.89 (< 5), indicating poor diagnostic value. We observed that the different distribution of samples in CS3 could be a contributing factor to the different diagnostic value of CS3 to stage C. In Angelieri's study, 82.6% of patients were in stage C and 17.4% were in stage B, while in our study, 58.3% of patients were in stage C and 41.7% were in stage B. This difference may be due to differences in ethnicity. The distribution of samples from the Asian population in CS3 is similar to that reported by Jang et al. [17]. Although the positive LHR of CS3 to stage C indicates poor diagnostic value in our study, we believe that CBCT is not necessary in patients with CS3, because both stage B and stage C indicate that the midpalatal suture is not yet fully fused.

## Conclusion

The results showed a high correlation between CVMS and MPSMS. Specifically, CS12 of CVMS presented a high diagnostic value for stage AB of MPSMS, making it a reliable indicator. The CS4 and CS56 stages of CVMS have moderate diagnostic value for the C and DE stages of MPSMS, respectively. In clinical practice, patients with CS4 and CS56 CVMS are recommended undergo a CBCT scan to assess the maturation stages of the midpalatal suture.

## Data Availability

The datasets used and/or analysed during the current study available from the corresponding author on reasonable request.
